# Evaluation of albendazole efficiency and complications in patients with pulmonary hydatid cyst

**DOI:** 10.1093/icvts/ivab259

**Published:** 2021-09-29

**Authors:** Yener Aydin, Ali Bilal Ulas, Ilker Ince, Asli Kalin, Fatma Kesmez Can, Betul Gundogdu, Kamber Kasali, Bugra Kerget, Yasemin Ogul, Atilla Eroglu

**Affiliations:** Department of Thoracic Surgery, Faculty of Medicine, Ataturk University, Erzurum, Turkey; Anesthesiology Clinical Research Office, Faculty of Medicine, Ataturk University, Erzurum, Turkey; Department of Thoracic Surgery, Faculty of Medicine, Ataturk University, Erzurum, Turkey; Anesthesiology Clinical Research Office, Faculty of Medicine, Ataturk University, Erzurum, Turkey; Department of Anesthesiology and Reanimation, Faculty of Medicine, Ataturk University, Erzurum, Turkey; Nuffield Department of Primary Care Health Sciences, University of Oxford, Oxford, UK; Department of Infectious Diseases and Clinical Microbiology, Faculty of Medicine, Ataturk University, Erzurum, Turkey; Department of Pathology, Faculty of Medicine, Ataturk University, Erzurum, Turkey; Department of Bioistatistics, Faculty of Medicine, Ataturk University, Erzurum, Turkey; Department of Pulmonary Diseases, Faculty of Medicine, Ataturk University, Erzurum, Turkey; Department of Medicinal Biochemistry, Regional Training and Research Hospital, Health Sciences University, Erzurum, Turkey; Department of Thoracic Surgery, Faculty of Medicine, Ataturk University, Erzurum, Turkey

**Keywords:** Albendazole, Hydatid cyst, Lung, Complication, Reliability

## Abstract

**OBJECTIVES:**

This study investigated the efficacy and complications of albendazole use after surgery in patients with pulmonary hydatid cysts.

**METHODS:**

One hundred fifty-three consecutive patients who met the study criteria out of 215 patients who received prophylaxis with albendazole after surgery for isolated pulmonary hydatid cysts in our clinic between January 2011 and December 2020 were analysed retrospectively.

**RESULTS:**

Eighty-six out of 153 (56.2%) of cases were male and 67 (43.8%) were female. The average age was 24.6 ± 17.4 (between 3 and 71 years), 76 of them (49.7%) were 18 years old and younger, while 77 (50.3%) were adults. All cases were approached transthoracically and a total of 170 operations were performed on the 153 cases. Fever, weakness and dizziness were reported in only one patient who was given albendazole treatment. A partial increase in liver enzymes was observed in 16 cases (10.5%) after albendazole treatment. Mild leukopoenia and neutropenia were observed in only one of the cases. In 1 case, a second operation was performed 30 months later due to recurrence. Albendazole treatment was not required to be discontinued in any of the cases. Mortality was not observed in any of the cases. Factors such as mean age, cyst size and hospitalization period did not have a statistically significant effect on any changes in liver enzymes tests following albendazole therapy (*P* > 0.05).

**CONCLUSIONS:**

Albendazole treatment can safely be used for postoperative prophylaxis in patients with pulmonary hydatid cysts in a controlled manner without causing serious complications.

**Subj collection:**

152.

**Figure:**
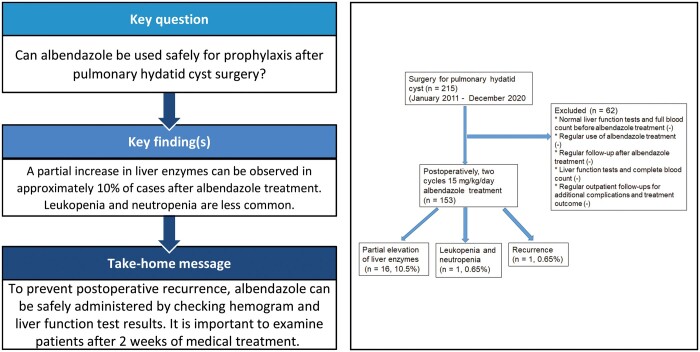

## INTRODUCTION

Hydatid cyst disease is a parasitic disease that is common in countries where agriculture and animal husbandry are common and has a wide geographical distribution in the world. This disease is caused by the metacestode stage larva of Echinococcus granulosus, a flatworm. It is transmitted to humans through animals that are part of its life cycle and through dog faeces in particular [1].

Although hydatid cysts can develop in every body organ, the liver is the most commonly affected location in adults (60–80%) followed by the lungs (10–30%). In children, the most affected organ is the lung [2, 3].

Excluding cases where chemotherapy regimens are required, the definitive treatment of pulmonary hydatid cyst is surgery. Although it has been reported that very small cysts can be eliminated spontaneously, surgery is still the most effective treatment method for hydatid cysts. Since the use of benzimidazole has seen some curative results, albendazole and mebendazole have been used in the treatment of hydatid cysts since the 1980s. Most centres, including our clinic, do not recommend preoperative medical treatment for pulmonary hydatid cysts. Medical treatment is mostly applied in the postoperative period [4, 5]. In this study, the effectiveness and complications of albendazole treatment applied after pulmonary hydatid cyst surgery were investigated.

## MATERIALS AND METHODS

### Study design

This retrospective study was conducted at Ataturk University Medical Faculty, Department of Thoracic Surgery. The study protocol was approved by the Institutional Review Board for Human Subjects Research and Ethics Committee of Ataturk University Medical Faculty. The study was conducted in accordance with the principles of the Declaration of Helsinki.

### Study subjects

Routine practice in our clinic in managing patients with pulmonary hydatid cysts is as follows: albendazole treatment is started upon discharge in patients with normal post-operative liver function tests and full blood count and based on their weight (15 mg/kg/day). The patient is then invited back for review 15 days later to repeat liver function tests and a full blood count, as well as have a chest X-ray radiography. Following this review, the drug is discontinued for 10 days and a second course of treatment is started for another 15 days at the end of which the patient is reviewed once again in clinic. At this stage, another set of liver function tests and full blood count are performed as well as a repeat chest X-ray radiography. In patients with bilateral hydatid cysts, albendazole prophylaxis is initiated after the surgical treatment of the contralateral lung cyst.

Patients with isolated pulmonary hydatid cysts (unilateral or bilateral), who underwent surgical treatment for hydatid cysts and who received albendazole treatment after surgery, were included in the study. Other criteria for inclusion in the study were: normal liver function tests and full blood count before albendazole treatment, completion of full treatment with albendazole, regular liver function tests and full blood counts after each course of albendazole and regular follow-ups for additional complications and treatment results. Cases that did not meet these conditions were not included in the study.

Among 215 patients who were operated on for unilateral or bilateral isolated pulmonary hydatid cysts between January 2011 and December 2020, 153 patients who met the study criteria were evaluated retrospectively. The age and gender of the cases, localization, number, size and the intact or ruptured status of the cysts, liver enzyme tests, full blood counts and any complications observed in the patients were reviewed.

All cases were approached transthoracically and a total of 170 operations were performed on 153 cases (16 cases were bilateral and 1 case recurred). While cystotomy and capitonage were performed in 164 of 170 surgeries, wedge resection was performed thoracoscopically in 5 cases in the form of solitary pulmonary nodules. As a result of frozen-section examinations, hydatid cysts were detected in these cases. In 1 case, a left upper lobectomy was performed due to a destroyed lung. Histopathologically, hydatid cysts were detected in all cases. Postoperatively, 15 mg/kg/day albendazole treatment was given to the patients in 2 cycles of 15 days each. A drug-free rest period of 10 days was applied between the 2 cycles.

### Statistical analysis

Analyses were made with IBM SPSS 20 statistical analysis program. Data were presented as mean, standard deviation, median, minimum, maximum, percentage and number. The normal distribution of continuous variables was analysed by the Shapiro–Wilk-*W* test and Kolmogorov–Smirnov test. In the comparisons between 2 independent groups, the independent samples *t*-test was used when the normal distribution condition was met and the Mann–Whitney *U*-test was used if it was not. In 2 × 2 comparisons between categorical variables, if the expected value was >5, the Pearson chi-square test was used, if the expected value was between 3 and 5, the chi-square Yates test was used and if the expected value was <3, the Fisher's exact test was used. Statistical significance level was taken as *P* < 0.05.

## RESULTS

In our study, 86 (56.2%) of the cases were male and 67 (43.8%) were female. The average age was 24.6 ± 17.4 (between 3 and 71 years), 76 (49.7%) were 18 years old and younger, while 77 (50.3%) were adults. While the hydatid cyst in 127 (83%) of the cases was solitary (17%), 26 cases were had multiple pulmonary cysts. While 16 of the multiple hydatid cysts were bilaterally located, 10 cases had >1 hydatid cyst in 1 lung. The distribution of hydatid cysts according to localization was as follows: 73 (41.2%) cases in the right lower lobe, 51 (28.8%) cases in the left lower lobe, 24 (13.6%) cases in the right upper lobe, 23 (13.0%) cases in the left upper lobe and 6 (3.4%) cases in the right middle lobe. The size of the cysts was 7.27 ± 3.0 cm (range 2–22 cm). Hydatid cysts were ruptured in 70 (45.8%) cases and intact in 83 (54.2%) cases.

Postoperatively, empyema was observed in 2 cases, a prolonged air leak in 1 case and wound infection in 1 case. Fever, weakness and dizziness were reported in only one patient among the patients who were given albendazole treatment.

**Figure 1:**
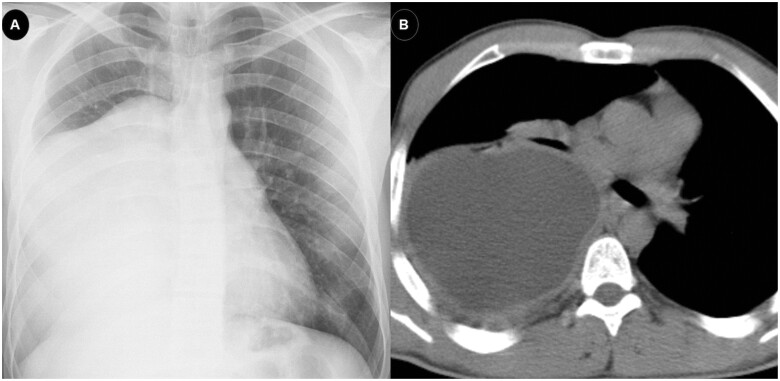
Posteroanterior chest X-ray (**A**) and axial thorax computed tomography scan (**B**) of a 22-year-old male patient who had a giant hydatid cyst of ∼44 mm × 123 mm × 185 mm in the right lower lobe and recurred later.

Partial elevation of liver enzymes occurred in 16 (10.5%) patients treated with albendazole (reference range; gamma-glutamyl transferase: 1–38 U/l, aspartate aminotransferase: 1–35 U/l, alanine aminotransferase: 1–35 U/l, lactate dehydrogenase: 1–247 U/l). Mild leukopoenia and neutropenia were observed in only one (0.65%) of the cases (leucocyte: 3340, neutrophil: 28.4%; reference range 3900–10 800 for leucocyte and 42.3–77.7% for neutrophil). Only one case required a second surgery 30 months after the first surgery. This was due to cyst recurrence (Fig. 1). Albendazole treatment was not required to be discontinued in any of the cases. Mortality was not observed in any of the cases. The mean postoperative hospital stay was 6.1 ± 3.57 days (between 2 and 30 days) (Table 1).

**Table 1:** Patient characteristics

	Count	Column *N* (%)
Cyst localization		
Right lower lobe	56	36.6
Right lower, right middle lobe	3	2.0
Right lower, right upper lobe	1	0.7
Right lower, left lower lobe	7	4.6
Right lower, left lower, left upper lobe	2	1.3
Right lower, left upper lobe	4	2.6
Right middle lobe	3	2.0
Right upper lobe	20	13.0
Right upper, left lower lobe	1	0.7
Right upper, left upper lobe	2	1.3
Left lower lobe	40	26.0
Left lower, left upper lobe	1	0.7
Left upper lobe	13	8.5
Number of cysts		
Multiple	26	17.0
Solitary	127	83.0
State of cysts		
Intact	83	54.2
Ruptured	70	45.8
Surgery		
Cystotomy and capitonnage	164	96.5
Thoracoscopic wedge resection	5	2.9
Left upper lobectomy	1	0.6
Liver enzymes		
ALT < ×2 the normal range	7	4.6
AST < ×2 and ALT < ×4 the normal range	2	1.3
LDH, AST and ALT < ×2 the normal range	6	3.9
GGT < ×2, AST and ALT < ×3 the normal range	1	0.7
Normal	137	89.5
Full blood count		
Leukopoenia	1	0.7
Normal	152	99.3
Complication		
Empyema	2	1.3
Fever, weakness, dizziness	1	0.7
Prolonged air leak	1	0.7
Wound infection	1	0.7
No	148	96.7
Recurrence		
Yes	1	0.7
No	152	99.3
Cyst localization		

ALT: alanine aminotransferase; AST: aspartate aminotransferase; GGT: gamma-glutamyl transferase; LDH: lactate dehydrogenase.

There was no statistically significant difference between genders in the distribution of deranged liver enzyme tests, leucocyte counts, presence or absence of complications and recurrence (*P* > 0.05). Similarly, factors such as mean age, cyst size and hospitalization period did not have a statistically significant effect on any changes in liver enzymes tests following albendazole therapy (*P* > 0.05).

## DISCUSSION

Our study consisted of 153 patients who were operated on for an isolated pulmonary hydatid cyst and were administered postoperative prophylaxis with albendazole. In the laboratory tests after albendazole treatment, a partial increase in liver enzymes was observed in 10.5% of the cases and leukopoenia in 1 case. Furthermore, fever, weakness and dizziness were reported in only one patient who was given albendazole treatment and recurrence of the hydatid cyst was observed in 1 patient despite prophylaxis.

The general treatment approach for all hydatid cysts in the body is with open surgery. One of the most important problems in hydatid cyst surgery is recurrence seen in ∼25% of the patients [6]. Re-surgery after recurrence is associated with increased operative morbidity and mortality. Surgical intervention may sometimes be required due to the development of complications in patients who receive only medical treatment. It has been shown that albendazole treatment is highly effective in preventing recurrences, and the recurrence rate is significantly higher in patients not taking albendazole [6–9].

Albendazole treatment does not have a definite standard dose and duration [10–12]. The optimal duration of treatment is recommended as 3–6 months, but it can be extended as long as there are no side effects [11–13]. Studies have reported that the treatment period of 6–8 months will be sufficient for small and newly formed cysts, and the treatment period of cysts larger than 5 cm, multiple cysts and multiorgan involvement can be extended to 12–20 months [12, 14]. In our study, Albendazole treatment was administered postoperatively in 2 equally divided doses with 15 mg/kg/day. After 15 days of treatment, drug treatment was interrupted for 10 days. Drug treatment was given again for 15 days after the break. Recurrence was observed in only one of our cases. Two cycles of albendazole prophylaxis were not sufficient to prevent recurrence in this case with a giant cyst. It may be beneficial to extend the duration of albendazole treatment in cases with such giant cysts and a high risk of recurrence.

Albendazole is well tolerated by most patients. However, the most common side effects associated with albendazole use are headache and mild-to-moderate increase in liver enzymes in 10–20% of patients [6, 10, 11, 15]. Aminotransferase elevation may develop due to drug toxicity or elimination of the parasite. Although a transient increase in liver enzymes is common, little evidence of albendazole-induced liver damage has been reported in the literature [16]. Although albendazole is mainly metabolized in the liver, most of the cases in which liver enzyme elevations were reported had mild or moderate elevations even after prolonged administration and returned to normal after discontinuation of therapy [16].

Some other side effects of albendazole include abdominal pain, nausea, vomiting and fever. A small number of patients may experience a hypersensitivity reaction to the drug such as urticaria and itching. There are reports of alopecia and telogen effluvium as rare side effects of albendazole treatment in the literature. Fortunately, these complications usually return to normal after treatment is complete [11]. The frequency of serious side effects is highest when albendazole is administered in high doses for long periods [10]. Other serious side effects include leukopoenia, anaemia, thrombocytopaenia and pancytopenia, and patients with pre-existing liver damage or dysfunction are at the highest risk of developing these conditions. The most likely mechanism of albendazole-induced myelosuppression is β-tubulin inhibition of the drug that affects the host's microtubules [11]. In our study, a partial increase in liver enzymes was observed in 10.5% of the patients who received albendazole treatment. Mild leukopoenia and neutropenia were observed in only one of the cases. Fever, weakness and dizziness were reported in only one patient who was given albendazole treatment. Albendazole treatment was not discontinued in any of the patients, and liver functions and leucocyte levels returned to normal after treatment.

Liver function tests and complete blood count should be performed at the beginning of a 28-day cycle and every 2 weeks during treatment because of the risk of pancytopenia and anaemia. Patients with a predisposition to leukopoenia and pancytopenia, such as those who have recently received chemotherapy, should be closely monitored due to the risk of myelosuppression due to albendazole. Patients with liver disease require more frequent monitoring as the drug is mainly metabolized by the liver [11, 15].

The reason why there was no complaint of headache in our patients may be that analgesic and anti-inflammatory therapy were given together as albendazole treatment was given postoperatively. The reason for the absence of abdominal pain may be explained by the analgesic treatment given and the absence of any abdominal cysts that could be ruptured with treatment due to the inclusion of only patients with pulmonary hydatid cysts.

The clinical course differs according to the location of the hydatid cyst in the liver or lung. Albendazole is widely used as the primary treatment for hydatid disease of the liver and successful results have been reported [8, 9]. However, pulmonary hydatid cyst rupture can result in suppuration leading to abscess formation. For this reason, we do not find it safe to use only medical therapy in pulmonary hydatid cyst control. Even if the parasite in the lung is eliminated, membrane debris cause recurrent pulmonary infections [17]. Usluer *et al.* [4] examined the effect of preoperative administration of albendazole on the cuticular membrane in pulmonary hydatid cysts. They reported that albendazole treatment may cause perforation by reducing the tensile strength of cuticular membranes in pulmonary hydatid cysts.

### Limitations

One of the main limitations of this study is that it is a retrospective study. Another issue is that the administration of albendazole along with postoperative analgesic and anti-inflammatory treatment may have hidden the symptoms of patients with possible headache and abdominal pain.

## CONCLUSIONS

In conclusion, we think that albendazole treatment should be given as prophylaxis after surgery in patients with pulmonary hydatid cysts. As such, both the rupture of the pulmonary hydatid cyst due to medical treatment is prevented and a shorter and lower dose course of prophylaxis is required providing effective results. In addition, having a 10-day break after the first 2-week course of albendazole treatment is helpful in allowing any slight increase in liver enzymes to normalize, and there be able to complete the full treatment with fewer complications. Although an increase in liver enzymes and leukopoenia can be seen in patients, these are generally not severe and do not require discontinuation of albendazole therapy.

**Conflict of interest**: none declared.

### Author contributions

**Yener Aydin:** Conceptualization; Data curation; Formal analysis; Investigation; Methodology; Resources; Writing—original draft; Writing—review & editing. **Ali Bilal Ulas:** Conceptualization; Data curation; Investigation; Methodology; Writing—original draft. **Ilker Ince:** Conceptualization; Investigation; Methodology; Resources; Visualization; Writing—original draft. **Asli Kalin:** Methodology; Writing—original draft; Writing—review & editing. **Fatma Kesmez Can:** Conceptualization; Investigation; Methodology; Resources. **Betul Gundogdu:** Conceptualization; Data curation; Methodology. **Kamber Kasali:** Formal analysis; Software; Supervision. **Bugra Kerget:** Resources; Software; Visualization; Writing—original draft. **Yasemin Ogul:** Supervision; Writing—original draft; Writing—review & editing. **Atilla Eroglu:** Investigation; Methodology; Writing—review & editing.

### Reviewer information

Interactive CardioVascular and Thoracic Surgery thanks Olgun Kadir ARIBAŞ, Frank A. Baciewicz Jr and the other, anonymous reviewer(s) for their contribution to the peer review process of this article.
